# Clinical predictors of hypoxic pneumonia in children from the Eastern Highlands Province, Papua New Guinea: secondary analysis of two prospective observational studies

**DOI:** 10.1016/j.lanwpc.2024.101052

**Published:** 2024-03-27

**Authors:** Kathryn J. Britton, William Pomat, Joycelyn Sapura, John Kave, Birunu Nivio, Rebecca Ford, Wendy Kirarock, Hannah C. Moore, Lea-Ann Kirkham, Peter C. Richmond, Jocelyn Chan, Deborah Lehmann, Fiona M. Russell, Christopher C. Blyth

**Affiliations:** a Wesfarmers Centre of Vaccines and Infectious Diseases, Telethon Kids Institute, The University of Western Australia, Nedlands, Western Australia, Australia; b School of Medicine, The University of Western Australia, Nedlands, Western Australia, Australia; c Infection and Immunity Unit, Papua New Guinea Institute of Medical Research, Goroka, Eastern Highlands, Papua New Guinea; d School of Population Health, Curtin University, Perth, Western Australia, Australia; e Centre for Child Health Research, The University of Western Australia, Nedlands, Western Australia, Australia; f Infection and Immunity, Murdoch Children's Research Institute, Melbourne, Victoria, Australia; g Department of Paediatrics, The University of Melbourne, Melbourne, Victoria, Australia; h Department of Paediatrics, Centre for International Child Health, The University of Melbourne, Melbourne, Victoria, Australia; i Department of Infectious Diseases, Perth Children's Hospital, Nedlands, Western Australia, Australia; j Department of Microbiology, PathWest Laboratory Medicine, QEII Medical Centre, Nedlands, Western Australia, Australia

**Keywords:** Pneumonia, Child, Hypoxaemia, Clinical signs, Papua New Guinea

## Abstract

**Background:**

Pneumonia is the leading cause of death in young children globally and is prevalent in the Papua New Guinea highlands. We investigated clinical predictors of hypoxic pneumonia to inform local treatment guidelines in this resource-limited setting.

**Methods:**

Between 2013 and 2020, two consecutive prospective observational studies were undertaken enrolling children 0–4 years presenting with pneumonia to health-care facilities in Goroka Town, Eastern Highlands Province. Logistic regression models were developed to identify clinical predictors of hypoxic pneumonia (oxygen saturation <90% on presentation). Model performance was compared against established criteria to identify severe pneumonia.

**Findings:**

There were 2067 cases of pneumonia; hypoxaemia was detected in 36.1%. The strongest independent predictors of hypoxic pneumonia were central cyanosis on examination (adjusted odds ratio [aOR] 5.14; 95% CI 3.47–7.60), reduced breath sounds (aOR 2.92; 95% CI 2.30–3.71), and nasal flaring or grunting (aOR 2.34; 95% CI 1.62–3.38). While the model developed to predict hypoxic pneumonia outperformed established pneumonia severity criteria, it was not sensitive enough to be clinically useful at this time.

**Interpretation:**

Given signs and symptoms are unable to accurately detect hypoxia, all health care facilities should be equipped with pulse oximeters. However, for the health care worker without access to pulse oximetry, consideration of central cyanosis, reduced breath sounds, nasal flaring or grunting, age-specific tachycardia, wheezing, parent-reported drowsiness, or bronchial breathing as suggestive of hypoxaemic pneumonia, and thus severe disease, may prove useful in guiding management, hospital referral and use of oxygen therapy.

**Funding:**

Funded by 10.13039/100004319Pfizer Global and the 10.13039/100000865Bill & Melinda Gates Foundation.

**Research in context**

**Evidence before this study**

Case management is a strategy used in resource-limited settings to identify children with severe disease most likely to benefit from in-patient treatment. Developed by the World Health Organization (WHO), the Integrated Management of Childhood Illness (IMCI) case management strategy was introduced into Papua New Guinea in 1999. The IMCI guidelines, revised in 2013, currently include two categories of pneumonia: “pneumonia” with fast breathing and/or chest indrawing, with home therapy using oral amoxicillin recommended, and “severe pneumonia”, pneumonia with any general danger sign, requiring referral to hospital and injectable therapy. WHO also provides recommendations on use of clinical signs to detect hypoxaemia when pulse oximetry is unavailable. These case definitions guide decisions on hospital admission, administration of antibiotics and oxygen therapy in PNG.

Given the many changes to lifestyle, healthcare access, antimicrobial use, and the childhood immunization programme seen over the past decade, both the *PNG treatment manual* and the IMCI case management strategy warrant re-examination. With pulse oximetry still not available in all health care facilities, use of these clinical definitions to predict hypoxia should be reconsidered.

**Added value of this study**

In children presenting for care with clinical pneumonia in PNG, hypoxia remains a common complication (36.1% of those with moderate to severe pneumonia), particularly in the very young. Using data from children aged less than 5 years presenting with pneumonia, we identified numerous predictors for hypoxaemia suitable for inclusion in a predictive model. Key predictors included central cyanosis, reduced breath sounds and nasal flaring or grunting. When combined, the developed predictive model had a sensitivity and specificity of 53% and 88% respectively (area under the ROC curve of 0.781), superior to existing definitions including the PNG severe pneumonia definition (sensitivity, 25%; specificity, 96%; AUC, 0.615), WHO severe pneumonia definition (sensitivity, 17%; specificity, 94%; AUC, 0.597) and WHO clinical signs to guide oxygen therapy (sensitivity, 45%; specificity, 87%; AUC 0.673).

**Implications of all the available evidence**

Using contemporary data, this study confirms the inadequacy of clinical signs for detecting hypoxia in childhood pneumonia in PNG. Incorporating additional predictors improves the sensitivity and predictive value when compared with existing definitions including the PNG and WHO definitions. Despite this, the predictive algorithm however would still miss half of hypoxic children presenting with pneumonia. Efforts to ensure availability of pulse oximetry must be prioritized in all health care settings to ensure early detection of hypoxia in children with pneumonia and institution of oxygen therapy. Inclusion of the predictors and model into health care worker training may assist with timely recognition, appropriate management and hospital referral but should not replace routine pulse oximetry.

## Introduction

Pneumonia is the leading cause of death in young children globally, and the Asia–Pacific region reports the greatest number of cases in children each year.^1^^,^^2^ In Papua New Guinea (PNG), pneumonia is the most common cause of morbidity and mortality in children aged less than 5 years, accounting for approximately one in four hospitalizations.^3^^,^^4^ Hypoxia (typically defined as peripheral oxygen saturation less than 90%) is a common complication of childhood pneumonia, and an important indicator of severity. It occurs in approximately 40% of children presenting to hospitals in the PNG highlands with pneumonia and is associated with an increased risk of mortality.^5^, ^6^, ^7^, ^8^, ^9^ Oxygen therapy in children with hypoxic pneumonia can reduce mortality by approximately 20%; however, detecting hypoxaemia in settings where pulse oximetry is inconsistently available is challenging.^7^^,^^9^

Case management is a strategy used in resource-limited settings to identify children with severe disease most likely to benefit from in-patient treatment.^10^ Developed by the World Health Organization (WHO), the Integrated Management of Childhood Illness (IMCI) case management strategy was introduced into PNG in 1999. While the approach is based on guidelines developed and evaluated in PNG during the 1970s, implementation of the strategy in this country has not been widespread.^11^ The IMCI guidelines, revised in 2013, currently include two categories of pneumonia: “pneumonia” with fast breathing and/or chest indrawing, with home therapy using oral amoxicillin recommended, and “severe pneumonia”, pneumonia with any general danger sign, requiring referral to hospital and injectable therapy (Table 1).^12^ WHO also provides recommendations on use of clinical signs to detect hypoxaemia when pulse oximetry is unavailable.^13^

**Table 1:** Key classifications used within this paper.

Classification	Description
WHO pneumonia^8^	Cough and/or difficulty breathing plus fast breathing and/or chest indrawing.
WHO severe pneumonia^8^	Cough and/or difficulty breathing plus any danger sign.
WHO danger signs^8^	Inability to drink, persistent vomiting, convulsions, lethargy or unconsciousness, stridor in a calm child, severe malnutrition.
PNG mild pneumonia^11^	Cough and tachypnoea (>60/min if <2 months, >50/min if 2–12 months, >40/min if >12 months of age).
PNG moderate pneumonia^11^	Cough, tachypnoea (>60/min if <2 months, >50/min if 2–12 months, >40/min if >12 months of age), and chest indrawing.
PNG severe pneumonia^11^	Cough, tachypnoea (>60/min if <2 months, >50/min if 2–12 months, >40/min if >12 months of age), and chest indrawing plus any of the following: heart failure (pulse >160/min with hepatomegaly >2 cm below costal margin), cyanosis or restlessness, or inability to breastfeed/drink, or vomiting.
WHO clinical signs to guide oxygen therapy in absence of pulse oximeter^9^	Central cyanosis, inability to drink, severe chest indrawing, respiratory rate ≥70/min, grunting with every breath (in young infants), depressed mental status.

With a population of over nine million, PNG is a lower-middle-income country with one of the highest infant mortality rates in the Western Pacific region (35 deaths per 1000 live births in 2022).^14^ Pneumonia treatment is guided by the *PNG Standard Treatment Manual for Common Illnesses in Children* (hereafter referred to as the *PNG treatment manual*).^15^ Using clinical parameters, children presenting to a health-care facility are classified as having mild, moderate or severe pneumonia (Table 1). Following the principles of IMCI albeit using slightly different case definitions, this categorization guides decisions on hospital admission, administration of antibiotics and oxygen therapy. As per the *PNG treatment manual*, it is recommended that cases with moderate or severe pneumonia are admitted or referred to hospital.^15^

While case management approaches are utilized in many resource-poor countries and have been shown to reduce both overall and pneumonia-specific mortality, over-referral of children to hospital and the accurate recognition of hypoxaemia have both been identified as limitations of the approach.^16^ The clinical criteria used are deliberately sensitive to maximize the identification of cases requiring hospitalization and antibiotic therapy; however potentially unnecessary referral incurs a significant cost to the parent and limited benefit to the child.^17^ Furthermore, these definitions can overlap with the presentation of other diseases that do not require or require different antimicrobials, particularly in regions including PNG, where HIV, malnutrition, tuberculosis and malaria are endemic.^18^

While the predictive value of clinical signs in detecting hypoxic pneumonia has been poor in previous studies, it has not been reassessed in PNG in recent years.^7^^,^^9^ Given the many changes to lifestyle, healthcare access, antimicrobial use, and the childhood immunization programme seen over the past decade, both the *PNG treatment manual* and the IMCI case management strategy warrant re-examination. The objectives of this study are therefore to: i) describe the characteristics of children presenting with pneumonia to health care facilities in the Eastern Highlands Province (EHP); ii) identify clinical predictors of hypoxic pneumonia; and iii) evaluate current PNG guidelines for referral to hospital, as well as the WHO guidelines for management of childhood pneumonia, used in many resource-poor countries but not yet implemented widely in PNG.

## Methods

### Study design

We undertook a secondary analysis of data collected in two previously conducted observational studies.^5^^,^^6^^,^^19^ Study 1 enrolled children 0–4 years of age to determine the aetiology of clinically defined moderate or severe pneumonia (and/or meningitis). Recruitment began in January 2013, prior to the national rollout of the 13-valent pneumococcal conjugate vaccine (PCV13) and ceased in January 2016.^6^ Study 2, a prospective cohort study (April 2016–January 2019), formed part of a multi-country evaluation of the PCV13 coverage required for indirect protection against vaccine-type pneumococcal carriage.^19^ Both studies were conducted at Eastern Highlands Provincial Hospital and surrounding urban outpatient clinics, in the Eastern Highlands Province of PNG, employing the same eligibility criteria for enrolment.

### Study procedures

In both studies, children 0–4 years of age presenting to health care facilities with clinically defined moderate or severe pneumonia (as per the *PNG treatment manual*^15^) were eligible for enrolment (Table 1). Children with severe congenital abnormalities, hospitalization in the prior 14 days and/or those enrolled as a pneumonia case in the past 30 days were excluded from both studies. The presence of severe congenital abnormalities was determined by health care workers at the time of presentation. After obtaining informed consent, clinically trained research staff obtained a medical history, conducted a full physical examination (including pulse oximetry), and collected laboratory specimens.

All children had anthropometry performed with malnutrition defined as weight-for-age Z score <−2 on WHO growth charts. Vaccination status was determined using parent-held childhood immunization records. Children were considered adequately vaccinated against PCV13 and/or diphtheria, tetanus, whole-cell pertussis, hepatitis B and *Haemophilus influenzae* type b vaccine (DTPw-HepB-Hib) if they had received at least two doses between 0 and 11 months of age; and for those children aged 12 months and older, if they had received one or more doses after their first birthday. Vaccination at any time prior to presentation was considered a valid dose.^5^

Pneumonia treatment and decision to admit to hospital were at the discretion of the hospital medical staff. All admitted cases were reassessed daily during hospitalization.

### Outcomes of interest

The primary outcome measure for this analysis was hypoxic pneumonia, defined as an oxygen saturation of <90% in a child with pneumonia, measured in room air using a LifeBox Pulse Oximeter (Acare Technology Co. Ltd, Taiwan). Secondary outcomes were admission to hospital, bacteraemia, and in-hospital mortality. A blood culture was considered a true positive (bacteraemia) when at least one of the following microorganisms was isolated: *H. influenzae*, *Streptococcus pneumoniae*, beta-haemolytic Streptococci, *Staphylococcus aureus* and Enterobacteriaceae spp. The following organisms were regarded as contaminants: other *Staphylococcus* spp., other streptococcal spp., diphtheroids, *Bacillus* spp. and *Micrococcus* spp. A blood culture was considered negative if no growth was reported after 5 days of incubation at 35 °C using an automated blood culture system (BD, Franklin Lakes, NJ) or when unavailable, incubation at 35–37 °C with daily subculture.

### Statistical analysis

All study data were entered into a single database (FileMaker Pro 11; DB services, Indianapolis, US). Statistical analyses were performed with Stata 16 (Stata Corp., College Station, TX). Categorical data were described as numbers and percentages with comparison by χ^2^ test, or Fisher's exact test where appropriate. Continuous variables were reported as median and interquartile range (IQR) and compared by the Wilcoxon rank-sum test. Unadjusted and multivariable logistic regression analyses were performed to assess associations between predictive variables and hypoxic pneumonia.

For the initial multivariable analysis, the full model was developed using all explanatory variables. Models were developed for each age group (children <2 months, 2–11 months, and 12–59 months), and a further model developed for all children across both studies. Variables were removed using backward selection using a level of significance of *p* ≥ 0.1 utilizing the *stepwise* function in STATA. All removed variables were subsequently reinserted to assess for model improvement. A further model was developed by combining all significant variables from any of the three age-specific models. This was run for all three age groups, and all children with age group included as a categorical variable. Odds ratios (OR) and 95% confidence intervals (CI) are presented.

We estimated the validity of these models to predict hypoxaemia by calculating the sensitivity, specificity, positive and negative predictive values, positive and negative likelihood ratios, and area under the receiver operator characteristic (ROC) curve (AUC) from the predictive models fitted values. Utilizing the dataset, we compared the performance to predict hypoxic pneumonia against established definitions: PNG definition of severe pneumonia from the *PNG Treatment Manual*, WHO criteria for severe pneumonia, and WHO clinical signs for detection of hypoxaemia.^12^^,^^13^^,^^15^

### Ethics statement

The Papua New Guinea Institute of Medical Research Institutional Review Board (IRB#1510), PNG Medical Research Advisory Committee (MRAC 15.19/16.09), The Royal Children's Hospital Human Resources Ethics Committee (HREC reference number 35249) and The University of Western Australia Human Research Ethics Committee (RA/4/1/7960) provided ethical approval. Parents/caregivers provided written informed consent at enrolment.

### Role of the funding source

Study 1 was funded by Pfizer Global (WS1801400) and Study 2 by the Bill & Melinda Gates Foundation (OPP1115490). The funders had no role in study design, data collection, data analysis, data interpretation, or writing of the report. The corresponding author had full access to all data in the study and had final responsibility for the decision to submit for publication.

## Results

During the periods of study (January 2013–January 2019), 2047 children were enrolled: 898 from Study 1, and 1149 from Study 2. From the total cohort, 885 (43.2%) were female, and 1250 (61.1%) were less than 12 months old. Approximately one-third of children were hospitalized (n = 623, 30.4%), and of these, 12 died (1.9%). Malnutrition was present in 424 children (20.9%) and 667 (34.2%) had received age-appropriate doses of PCV13 and DTPw-HepB-Hib vaccines.

Among those enrolled, 281 (13.7%) participants had severe pneumonia according to the PNG definition (Table 1). A total of 739 (36.1%) children had hypoxaemia on presentation with a decreasing trend across age groups (infants <2 months: 47.5%; infants 2–11 months: 39.2%; children 12 months and older: 30.2%; Table 2). On physical examination, crepitations were the most frequently observed clinical sign (98.3%), followed by bronchial breathing (68.5%), and reduced breath sounds (58.0%). Infants aged <2 months more often presented with central cyanosis compared to children in older age groups (20.8% in infants <2 months vs 12.8% in infants 2–11 months and 6.4% in children ≥12 months); however, were less commonly febrile. Parents frequently reported fever and fast breathing in all age groups. Compared to central cyanosis and/or apnoea observed by a health-care worker (HCW) on physical examination, parents under-reported these findings (Table 2).

**Table 2:** Characteristics and clinical features of the study population: children aged 0–59 months presenting with moderate or severe pneumonia to health facilities in the Eastern Highlands Province between 2013 and 2019.

Characteristics	Age <2 months (n = 99); n (%)	Age 2–11 months (n = 1151); n (%)	Age 12–59 months (n = 797); n (%)	Total (N = 2047); n (%)
**Demographic**				
Female sex	43 (43.4)	503 (43.7)	339 (42.5)	885 (43.2)
**Risk factors**				
Admitted in wet season^a^	43 (43.4)	440 (38.2)	328 (41.1)	881 (39.6)
<1 h travel time to Goroka Town	66 (66.7)	775 (67.3)	589 (73.9)	1430 (73.9)
Malnutrition^b^	18 (18.2)	215 (18.7)	195 (24.5)	424 (20.9)
Comorbid conditions^c^	2 (2.0)	5 (0.4)	10 (1.3)	17 (0.8)
Adequately vaccinated^d^	n/a	365 (31.7)	302 (37.9)	667 (34.2)
**Clinical history (parent reported)**				
Fever	79 (79.8)	1086 (94.3)	757 (95.0)	1922 (93.9)
Fast breathing	99 (100.0)	1115 (97.0)	769 (96.5)	1983 (96.9)
Apnoea or cyanosis	12 (12.1)	77 (6.7)	24 (3.0)	113 (5.5)
Difficulty feeding or drinking	27 (27.3)	204 (17.8)	79 (9.9)	310 (15.2)
Vomiting	31 (31.1)	196 (17.1)	80 (10.0)	307 (15.0)
Drowsiness	5 (5.0)	81 (7.1)	51 (6.4)	137 (6.7)
Irritability	38 (38.4)	563 (49.0)	293 (36.8)	894 (43.7)
Convulsions or seizures	2 (2.0)	28 (2.4)	7 (0.9)	37 (1.8)
**Physical examination (HCW assessed)**				
Fever (≥38 °C)	15 (15.0)	382 (33.2)	302 (37.9)	699 (34.1)
Age-specific tachycardia^e^	25 (25.5)	247 (21.6)	210 (26.4)	482 (23.7)
Central cyanosis	20 (20.8)	143 (12.8)	50 (6.4)	213 (10.7)
Apnoea observed	29 (30.2)	223 (19.5)	89 (11.2)	341 (16.8)
Nasal discharge	28 (28.3)	653 (56.8)	530 (66.5)	1211 (59.2)
Severe lower chest indrawing	21 (21.1)	178 (15.5)	40 (5.0)	239 (11.7)
Nasal flaring or grunting	16 (16.7)	165 (14.8)	55 (6.9)	236 (11.8)
Delayed capillary refill (<3 s)	7 (7.1)	49 (4.3)	22 (2.8)	78 (3.8)
Clinical dehydration^f^	9 (9.1)	102 (8.9)	57 (7.1)	16 (8.2)
Abdominal distention	2 (2.0)	17 (1.5)	97 (12.2)	116 (5.7)
Pallor	4 (4.1)	13 (1.1)	17 (2.1)	34 (1.7)
Stridor	4 (4.1)	37 (3.3)	21 (2.7)	62 (3.1)
Wheeze	16 (16.5)	289 (25.5)	189 (23.9)	494 (24.5)
Crepitations	92 (94.8)	1126 (98.2)	778 (98.7)	1996 (98.3)
Bronchial breathing	68 (71.6)	828 (73.3)	480 (61.1)	1376 (68.5)
Reduced breath sounds	68 (70.1)	693 (60.8)	412 (52.3)	1173 (58.0)
Hepatomegaly	1 (1.0)	42 (3.7)	18 (2.3)	61 (3.0)
Decreased consciousness	0	6 (0.5)	4 (0.5)	10 (0.5)
**Diagnosis and Outcomes**				
PNG-defined moderate pneumonia^g^	70 (70.7)	942 (81.8)	754 (94.6)	1766 (86.3)
PNG-defined severe pneumonia^g^	29 (29.3)	209 (18.2)	43 (5.4)	281 (13.7)
Hypoxaemia (O_2_ saturation <90%)	47 (47.5)	451 (39.2)	241 (30.2)	739 (36.1)
Admitted to hospital	61 (61.6)	419 (36.4)	143 (17.9)	623 (30.4)
Bacteraemia^h^	0	25 (2.3)	24 (3.2)	49 (2.5)
In-hospital death	1 (1.6)	9 (2.1)	2 (1.4)	12 (1.9)

HCW: Healthcare worker.

Denominators vary due to missing data.

a Wet season refers to period from December to April inclusive, dry season refers to period from May to November inclusive.

b Malnutrition defined as weight-for-age less than −2 z scores of median (WHO growth standards).

c Comorbid conditions: previously diagnosed conditions identified at the time of hospital presentation by parental report and review of handheld record.

d Adequately vaccinated: ≥2 doses of both DTPw-HepB-Hib and PCV13 in a child 2–11 months, ≥1 dose of both DTPw-HepB-Hib and PCV13 in a child ≥12 months. DTPw-HepB-Hib: diphtheria, tetanus, whole-cell pertussis, hepatitis B and *Haemophilus influenzae* type b vaccine. PCV13: 13-valent pneumococcal conjugate vaccine.

e Age-specific tachycardia: heart rate >160/min in a child <12 months, >120/min in a child aged 12 months to <5 years.^20^

f Signs of clinical dehydration include dry mucous membranes and/or reduced skin turgor.

g PNG-defined moderate pneumonia: cough, tachypnoea (respiratory rate ≥60/min if <2 months, ≥40/min if ≥2 months) and lower chest wall indrawing. PNG-defined severe pneumonia: moderate pneumonia plus heart failure (pulse >160/min with hepatomegaly >2 cm below costal margin), or cyanosis or restlessness, or inability to breastfeed/drink or vomiting.

h Bacteraemia: *Haemophilus influenzae*, *Streptococcus pneumoniae*, beta-haemolytic Streptococci, *Staphylococcus aureus* or Enterobacteriaceae spp. isolated from a blood culture.

Hypoxaemic children of all ages presented more frequently with central cyanosis, HCW-observed apnoea, severe lower chest wall indrawing, nasal flaring or grunting, bronchial breathing, and reduced breath sounds than non-hypoxaemic children. Parents reported apnoea or cyanosis, and difficulty feeding or drinking significantly more frequently among hypoxaemic children (Table 3).

**Table 3:** Characteristics and clinical features of hypoxaemic vs non-hypoxaemic children presenting with moderate or severe pneumonia to health facilities in the Eastern Highlands Province between 2013 and 2019, unadjusted analysis.

Characteristic	Age <2 months			Age 2–11 months			Age 12–59 months		
	Hypoxaemic n (%) (n = 47)	Non-hypoxaemic n (%) (n = 52)	Unadjusted OR (95% CI)	Hypoxaemic n (%) (n = 451)	Non-hypoxaemic n (%) (n = 700)	Unadjusted OR (95% CI)	Hypoxaemic n (%) (n = 241)	Non-hypoxaemic n (%) (n = 556)	Unadjusted OR (95% CI)
**Demographic**									
Female sex	17 (36.2)	26 (50.0)	0.57 (0.25–1.27)	203 (45.0)	300 (42.9)	1.09 (0.86–1.38)	111 (46.1)	228 (41.0)	1.23 (0.90–1.66)
**Risk factors**									
Admitted in wet season	18 (38.3)	25 (48.1)	0.67 (0.30–1.49)	165 (36.6)	275 (39.3)	0.89 (0.69–1.14)	84 (34.8)	244 (43.9)	**0.68 (0.50–0.94)**
<1 h travel time to Goroka Town^a^	31 (66.0)	35 (67.3)	0.94 (0.41–2.17)	318 (70.5)	457 (65.3)	1.27 (0.98–1.64)	192 (79.7)	397 (71.4)	**1.57 (1.09–2.26)**
Malnutrition^b^	10 (21.3)	8 (15.4)	1.48 (0.53–4.15)	91 (20.2)	124 (17.7)	1.17 (0.87–1.59)	64 (26.6)	131 (23.6)	1.17 (0.83–1.66)
Comorbid conditions^c^	2 (4.3)	0	–	1 (0.2)	4 (0.6)	0.39 (0.04–3.47)	3 (1.2)	7 (1.3)	0.98 (0.25–3.83)
Adequately vaccinated^d^	–	–		164 (36.4)	201 (28.7)	**1.42 (1.10–1.82)**	111 (46.1)	191 (34.3)	**1.63 (1.20–2.22)**
**Clinical history (parent reported)**									
Fever	40 (85.1)	39 (75.0)	1.90 (0.69–5.27)	428 (94.9)	658 (94.0)	1.19 (0.70–2.00)	234 (97.1)	523 (94.1)	2.11 (0.92–4.84)
Fast breathing	47 (100.0)	52 (100.0)	–	443 (98.2)	672 (96.1)	**2.22 (1.00–4.94)**	235 (97.5)	534 (96.0)	1.61 (0.65–4.03)
Apnoea or cyanosis	10 (21.3)	2 (3.8)	**6.76 (1.39–32.69)**	43 (9.6)	34 (4.9)	**2.06 (1.29–3.28)**	15 (6.2)	9 (1.62)	**4.03 (1.74–9.33)**
Difficulty feeding or drinking	20 (42.5)	7 (13.5)	**4.76 (1.78–12.74)**	105 (23.3)	99 (14.2)	**1.84 (1.35–2.49)**	39 (16.2)	40 (7.2)	**2.48 (1.55–3.98)**
Vomiting	19 (40.4)	12 (23.1)	2.26 (0.95–5.39)	89 (19.2)	107 (15.3)	**1.36 (1.00–1.86)**	32 (13.3)	48 (8.6)	**1.62 (1.01–2.61)**
Drowsiness	3 (6.4)	2 (3.8)	1.70 (0.27–10.67)	46 (10.2)	35 (5.0)	**2.15 (1.36–3.39)**	22 (9.1)	29 (5.2)	**1.82 (1.02–3.24)**
Irritability	14 (29.8)	24 (46.1)	0.49 (0.22–1.13)	242 (53.8)	321 (45.9)	**1.37 (1.08–1.74)**	117 (48.5)	176 (31.7)	**2.03 (1.49–2.77)**
Convulsions or seizures	2 (4.3)	0	–	12 (2.7)	16 (2.3)	1.16 (0.54–2.48)	1 (0.4)	6 (1.1)	0.38 (0.05–3.18)
**Physical examination (HCW assessed)**									
Fever (≥38 °C)	9 (19.1)	6 (11.5)	1.82 (0.59–5.56)	160 (35.5)	222 (31.7)	1.18 (0.92–1.52)	107 (44.4)	195 (35.1)	**1.48 (1.09–2.01)**
Age-specific tachycardia^e^	15 (31.9)	10 (19.6)	1.92 (0.76–4.84)	147 (32.7)	100 (14.4)	**2.88 (2.16–3.85)**	103 (42.9)	107 (19.3)	**3.14 (2.25–4.37)**
Central cyanosis	18 (40.0)	2 (3.9)	**16.3 (3.5–75.77)**	112 (25.7)	31 (4.6)	**7.25 (4.76–11.03)**	36 (15.2)	14 (2.6)	**6.84 (3.61–12.95)**
Apnoea observed	22 (47.8)	7 (14.0)	**5.63 (2.10–15.10)**	133 (29.7)	90 (12.9)	**2.84 (2.11–3.84)**	45 (18.7)	44 (7.9)	**12.66 (1.70–4.16)**
Nasal discharge	10 (21.3)	18 (34.6)	0.51 (0.21–1.26)	246 (54.5)	407 (58.3)	0.86 (0.67–1.09)	145 (60.2)	385 (69.2)	**0.67 (0.49–0.92)**
Severe lower chest indrawing	14 (30.4)	7 (13.5)	**2.81 (1.02–7.75)**	114 (25.3)	64 (9.2)	**3.35 (2.40–4.68)**	24 (10.0)	16 (2.9)	**3.72 (1.94–7.15)**
Nasal flaring or grunting	14 (30.4)	2 (4.0)	**10.5 (2.23–49.35)**	122 (27.8)	43 (6.3)	**5.67 (3.91–8.24)**	36 (14.9)	19 (3.4)	**4.93 (2.76–8.79)**
Delayed capillary refill (<3 s)	6 (12.8)	1 (1.9)	7.46 (0.86–64.49)	31 (6.9)	18 (2.6)	**2.8 (1.53–5.02)**	10 (4.1)	12 (2.2)	1.96 (0.83–4.60)
Clinical dehydration^f^	5 (10.6)	4 (7.7)	1.43 (0.36–5.67)	40 (8.9)	62 (8.9)	1.00 (0.66–1.52)	17 (7.0)	40 (7.2)	0.98 (0.54–1.76)
Abdominal distention	1 (2.1)	1 (1.9)	1.12 (0.07–18.24)	9 (2.0)	8 (1.1)	1.76 (0.67–4.60)	14 (5.8)	83 (14.9)	**0.35 (0.19–0.63)**
Pallor	3 (6.4)	1 (2.0)	3.41 (0.34–33.97)	8 (1.8)	5 (0.7)	2.51 (0.81–7.72)	6 (2.49)	11 (1.98)	1.26 (0.46–3.45)
Stridor	3 (6.5)	1 (1.9)	3.56 (0.36–35.46)	26 (5.9)	11 (1.6)	**3.81 (1.86–7.79)**	17 (7.1)	4 (0.7)	**10.35 (3.44–31.10)**
Wheeze	9 (19.6)	7 (13.7)	1.53 (0.52–4.50)	148 (33.0)	141 (20.6)	**1.90 (1.45–2.48)**	91 (37.9)	98 (17.8)	**2.81 (2.00–3.95)**
Crepitations	45 (95.7)	47 (94.0)	1.44 (0.23–9.00)	448 (99.6)	678 (97.4)	**5.95 (1.37–25.75)**	238 (99.6)	540 (98.4)	3.97 (0.50–31.48)
Bronchial breathing	41 (87.2)	27 (56.2)	**5.31 (1.90–14.87)**	362 (80.6)	466 (68.5)	**1.91 (1.44–2.54)**	191 (79.6)	289 (53.0)	**3.45 (2.42–4.93)**
Reduced breath sounds	38 (80.8)	30 (60.0)	**2.81 (1.12–7.07)**	343 (76.2)	350 (50.8)	**3.10 (2.38–4.04)**	181 (75.4)	231 (42.2)	**4.20 (3.00–5.89)**
Hepatomegaly	1 (2.1)	0	–	29 (6.5)	13 (1.9)	**3.59 (1.85–7.00)**	12 (5.0)	6 (1.1)	**4.77 (1.77–12.90)**
Decreased consciousness	0	0	–	4 (0.9)	2 (0.3)	3.12 (0.57–17.10)	2 (0.8)	2 (0.4)	2.31 (0.32–16.49)

HCW: Healthcare worker.

Denominators vary due to missing data. Bold values indicate statistical significance.

a Wet season refers to period from December to April inclusive, dry season refers to period from May to November inclusive.

b Malnutrition defined as weight-for-age less than −2 z scores of median (WHO growth standards).

c Comorbid conditions: previously diagnosed conditions identified at the time of hospital presentation by parental report and review of handheld record.

d Adequately vaccinated: ≥2 doses of both DTPw-HepB-Hib and PCV13 in a child 2–11 months, ≥1 dose of both DTPw-HepB-Hib and PCV13 in a child ≥12 months. DTPw-HepB-Hib: diphtheria, tetanus, whole-cell pertussis, hepatitis B and *Haemophilus influenzae* type b vaccine. PCV13: 13-valent pneumococcal conjugate vaccine.

e Age-specific tachycardia: heart rate >160/min in a child <12 months, >120/min in a child aged 12 months to <5 years.^20^

f Signs of clinical dehydration include dry mucous membranes and/or reduced skin turgor.

Independent predictors of hypoxaemia were initially identified separately for each age group (Supplementary Table 1). Central cyanosis on examination was the strongest predictor of hypoxaemia in all age groups: adjusted odds ratio [aOR] 12.29 (95% confidence interval [95% CI] 1.86–81.11) in infants aged <2 months, aOR 4.95 (95% CI 3.03–8.09) in infants aged 2–11 months and aOR 4.91 (95% CI 2.29–10.55) in children aged 12–59 months.

Due to the similarities between age-specific predictors, and our desired objective to create a simple prediction model for clinical use independent of age, a further model was developed combining all significant variables from any of the three age-specific models (Table 4). In this model, central cyanosis on examination (aOR 5.14; 95% CI 3.47–7.60), reduced breath sounds (aOR 2.92; 95% CI 2.30–3.71), nasal flaring or grunting (aOR 2.34; 95% CI 1.62–3.38), age-specific tachycardia (aOR 1.89; 95% CI 1.46–2.44), wheezing (aOR 1.76; 95% CI 1.36–2.26), parent-reported drowsiness (aOR 1.75; 95% CI 1.14–2.69) and bronchial breathing (aOR 1.42; 95% CI 1.09–1.85) were independently associated with hypoxaemia on presentation. Residing within 1 h of the hospital and being adequately vaccinated with PCV13 and DTPw-HepB-Hib were also independently associated with hypoxaemia (aOR 1.55; 95% CI 1.21–1.99, and aOR 1.94; 95% CI 1.52–2.46, respectively). Presenting in the wet season was protective against hypoxaemia (aOR 0.71; 95% CI 0.57–0.89).

**Table 4:** Multivariable analysis of risk factors and clinical features associated with hypoxic pneumonia in children presenting with moderate or severe pneumonia to health facilities in the Eastern Highlands Province between 2013 and 2019.

Potential predictors of hypoxaemia	Adjusted OR (95% CI)			
	Age <2 months	Age 2–11 months	Age 12–59 months	All ages
Age				
<2 months	–	–	–	2.32 (1.38–3.90)
2–11 months	–	–	–	1.35 (1.07–1.69)
12–59 months	–	–	–	1
Presented in wet season	0.52 (0.18–1.49)	0.82 (0.61–1.09)	0.56 (0.38–0.81)	0.71 (0.57–0.89)
Proximity to hospital (<1 h)	1.13 (0.35–3.59)	1.54 (1.12–2.11)	1.58 (1.02–2.47)	1.55 (1.21–1.99)
Adequately vaccinated^a^	–	1.81 (1.32–2.46)	2.05 (1.39–3.03)	1.94 (1.52–2.46)
Drowsiness (parent reported)	0.44 (0.01–14.59)	1.89 (1.08–3.32)	1.61 (0.79–3.28)	1.75 (1.14–2.69)
Irritability (parent reported)	0.46 (0.15–1.41)	1.08 (0.81–1.45)	1.51 (1.03–2.21)	1.18 (0.94–1.48)
Age-specific tachycardia^b^	1.40 (0.36–5.43)	1.92 (1.34–2.73)	1.90 (1.28–2.84)	1.89 (1.46–2.44)
Central cyanosis (on examination)	9.47 (1.57–56.96)	5.27 (3.25–8.56)	4.68 (2.21–9.91)	5.14 (3.47–7.60)
Nasal flaring or grunting	6.19 (0.94–40.93)	2.50 (1.59–3.93)	1.82 (0.91–3.63)	2.34 (1.62–3.38)
Wheezing	0.51 (0.10–2.61)	1.68 (1.21–2.34)	2.16 (1.42–3.30)	1.76 (1.36–2.26)
Bronchial breathing	3.87 (1.04–14.44)	1.06 (0.74–1.51)	1.92 (1.23–2.98)	1.42 (1.09–1.85)
Reduced breath sounds	1.66 (0.50–5.50)	2.76 (2.02–3.77)	3.39 (2.26–5.08)	2.92 (2.30–3.71)

a Adequately vaccinated: ≥2 doses of both DTPw-HepB-Hib and PCV13 in a child 2–11 months, ≥1 dose of both DTPw-HepB-Hib and PCV13 in a child ≥12 months. DTPw-HepB-Hib: diphtheria, tetanus, whole-cell pertussis, hepatitis B and *Haemophilus influenzae* type b vaccine. PCV13: 13-valent pneumococcal conjugate vaccine.

b Age-specific tachycardia: heart rate >160/min in a child <12 months, >120/min in a child aged 12 months to <5 years.^20^

Both approaches to model development (age-specific and combined) demonstrated similar abilities to predict hypoxaemia in children with moderate or severe pneumonia with reasonable sensitivity (ranging 44–74%) and high specificity (ranging 74–92%; Supplementary Table 2). Both models performed better in infants aged <2 months compared to the older age groups (age specific model: AUC = 0.849 in those <2 months compared to AUC = 0.772–0.799 in older children; combined model: AUC = 0.834 in those <2 months compared to AUC = 0.769–0.796 in older children).

The predictive ability of existing definitions (PNG severe pneumonia^15^; WHO severe pneumonia^12^; WHO clinical signs for detecting hypoxaemia^13^) were then applied to the dataset to assess their predictive abilities for hypoxic pneumonia (Table 5). The PNG definition of severe pneumonia had very high specificity (96%) but low sensitivity (25%). Similarly, the WHO definition of severe pneumonia also had high specificity (94%) but very low sensitivity (17%). Use of WHO signs to detect hypoxaemia provided an improvement in sensitivity (45%) at the cost of specificity (87%). The model with the greater AUC was the combined model developed on our dataset (AUC: 0.781, sensitivity 53%, specificity 88%), combining season of presentation, proximity to hospital, vaccination status, drowsiness, irritability, tachycardia, central cyanosis, nasal flaring or grunting, wheezing, bronchial breathing, or reduced breath sounds. This was followed by the WHO clinical signs to guide oxygen therapy (AUC: 0.673), the PNG severe pneumonia (AUC: 0.615) and WHO severe pneumonia definition (AUC: 0.597).

**Table 5:** Validity of the developed model and existing definitions for detecting hypoxaemia in children from the present study.

Model	Age group	Sensitivity	Specificity	PPV	NPV	LR+	LR−	AUC
Combined model (developed on this dataset)^a^	Overall	53%	88%	72%	75%	4.46	0.54	0.781
	0–2m	69%	87%	84%	74%	5.17	0.36	0.834
	2–11m	51%	88%	74%	73%	4.29	0.55	0.769
	1–5y	44%	92%	70%	79%	5.34	0.61	0.795
PNG severe pneumonia^11^	Overall	25%	96%	76%	69%	5.78	0.80	0.615
	0–2m	58%	84%	76%	69%	3.68	0.50	0.731
	2–11m	26%	95%	76%	67%	4.93	0.78	0.615
	1–5y	14%	97%	70%	73%	5.53	0.88	0.579
WHO severe pneumonia^8^	Overall	17%	94%	63%	66%	2.92	0.88	0.597
	0–2m	43%	86%	73%	64%	3.14	0.66	0.664
	2–11m	21%	90%	59%	64%	2.18	0.88	0.580
	1–5y	11%	98%	67%	72%	5.36	0.91	0.602
WHO clinical signs to guide oxygen therapy in absence of pulse oximeter^9^	Overall	45%	87%	66%	74%	3.45	0.63	0.673
	0–2m	59%	94%	90%	72%	9.85	0.44	0.762
	2–11m	47%	85%	67%	71%	3.09	0.62	0.675
	1–5y	31%	93%	67%	75%	4.62	0.74	0.648

PPV = Positive Predictive Value; NPV = Negative Predictive Value; LR+ = positive likelihood ratio (LR+ = sensitivity/(1 − specificity)); LR− = negative likelihood ratio (LR− = specificity/(1 − sensitivity)); AUC = the area under the ROC curve. A positive LR >10 and a negative LR <0.1 are considered to exert highly significant changes in probability, such as to alter clinical management.^21^

a Presented in wet season^b^, proximity to hospital (<1 h), adequately vaccinated^c^, history of drowsiness, history of irritability, tachycardia^e^, central cyanosis, nasal flaring or grunting, wheezing, bronchial breathing, reduced breath sounds.^b^Wet season refers to period from December to April inclusive, dry season refers to period from May to November inclusive.^c^Adequately vaccinated: ≥2 doses of both DTPw-HepB-Hib and PCV13 in a child 2–11 months, ≥1 dose of both DTPw-HepB-Hib and PCV13 in a child ≥12 months. DTPw-HepB-Hib: diphtheria, tetanus, whole-cell pertussis, hepatitis B and *Haemophilus influenzae* type b vaccine. PCV13: 13-valent pneumococcal conjugate vaccine.^d^Signs of clinical dehydration include dry mucous membranes and/or reduced skin turgor.^e^Age-specific tachycardia: heart rate >160/min in a child <12 months, >120/min in a child aged 12 months–4 years.^20^

## Discussion

Our study reports on hypoxaemia in children aged under 5 years with moderate to severe pneumonia presenting for health care in the EHP and shows that hypoxaemia is highly prevalent in this population. Our overall hypoxaemia prevalence estimate is consistent with that reported by Rahman et al. in a 2022 systematic review of hypoxaemia in children with pneumonia in low- and middle-income countries (36.1% in our study, vs 31% (95% CI 26–36) in the systematic review).^22^ Similarly, we found that the prevalence of hypoxaemia was lower among older children than younger children.^22^, ^23^, ^24^ We provide the latest data on hypoxaemia prevalence in this setting which is essential to inform clinical care and national efforts to scale up pulse oximetry and oxygen therapy.

Despite significant changes in lifestyle, healthcare access, antimicrobial use, and the childhood immunization programme, the clinical pattern of pneumonia observed in the present investigation corresponds with previous studies of PNG children, with fever, nasal discharge, and crepitations noted in nearly all patients.^25^^,^^26^ The age and sex distribution of the study population is also similar to that described previously in this setting, with a greater proportion of male patients and children under one year of age presenting for care.^26^^,^^27^ Younger children were also more likely to have severe disease.^27^

Cyanosis, a respiratory rate >90 per minute, grunting, and drowsiness have previously been identified as risk factors for hypoxaemia in children in the Southern Highlands of PNG.^28^ Along with cyanosis, hepatomegaly and longer duration of cough have been associated with severe hypoxaemia (SpO_2_ < 69%).^7^ Within the current study, cyanosis, grunting, and parent-reported drowsiness were similarly predictive of hypoxaemia, however hepatomegaly was not significantly associated with hypoxaemia when combined with other clinical signs. Presence of bronchial breath sounds (along with crepitations) has been identified as a useful predictor of hypoxaemia among children >12 months in Zambia,^29^ and several studies have reported a significant association of nasal flaring with hypoxaemia.^20^^,^^21^^,^^28^^,^^30^

Previous studies have demonstrated that detection of hypoxaemia using clinical signs is sub-optimal, and it is well established that pulse oximetry is the preferred approach to determining the requirement for and response to oxygen.^7^ However, despite the introduction of pulse oximeters into five PNG hospitals in 2004 with intended national expansion by 2020, they are still not routinely available for use in the highlands.^4^ Many clinical guidelines exist to predict pneumonia severity including hypoxaemia. Using the breadth of data available through prospective studies conducted in PNG we developed our own prediction model in an attempt to improve upon these existing algorithms and identify a subpopulation at great risk of being hypoxic. Our model was found to have high specificity (88%) yet inadequate sensitivity (53%), indicating only half the children with hypoxaemia would be identified. Despite this, our model outperforms the others we tested, with a sensitivity more than twice that of the PNG definition of severe pneumonia (24%), and nearly three times that of the WHO definition of severe pneumonia (17%). These findings show that the criteria for severe pneumonia in the *PNG treatment manual* is preferable for use in this setting compared with WHO-defined severe pneumonia which is recommended for use in all resource poor settings. However, none of the models examined are sensitive enough to detect hypoxaemia in children with PNG clinically defined moderate or severe pneumonia in any age group.

To our knowledge, this is the first assessment and comparison of the validity of current pneumonia case definitions in PNG children. The strengths of this study include the recruitment and collection of comprehensive clinical data from over 2000 children with pneumonia. The hypoxaemia prevalence reported in this paper is estimated from 6 years’ worth of data, and we adjusted for potential confounders to give robust estimates of the clinical predictors of hypoxic pneumonia. As limitations of the study, residual confounding, as suggested by both vaccination and proximity to the hospital being independent predictors of hypoxic pneumonia, remained despite adjustment and revision of the model. Recruitment was undertaken within Goroka, therefore potentially limiting the generalizability of the findings. Although we attempted to exclude severe congenital abnormalities, these were determined clinically so it is possible that less severe abnormalities (e.g., less severe non-cyanotic heart disease) may have missed, given the difficulty in detection in very young infants. Clinical predictors and the prevalence of hypoxaemia were estimated in children with moderate or severe pneumonia; therefore, these results are not necessarily applicable to all children with pneumonia, or other conditions that result in hypoxaemia. As risk factors such as breast feeding and smoke exposure were only available for cases enrolled in Study 2, these potential cofounders could not be used in the analysis.

In agreement with the literature, we conclude that signs and symptoms are unable to accurately detect hypoxia, and all health centers and remote nursing posts should be equipped with pulse oximeters. However, for the HCW without access to pulse oximetry, children under the age of five presenting with pneumonia and central cyanosis, reduced breath sounds, nasal flaring or grunting, age-specific tachycardia, wheezing, parent-reported drowsiness or bronchial breathing require immediate referral to hospital. Consideration of these signs as suggestive of hypoxaemic pneumonia, and thus severe disease, may prove useful in guiding management and use of oxygen therapy, a limited resource in this setting. Ongoing training of HCWs is critical to ensure they recognize the signs of severe disease, provide timely and appropriate management and refer to hospital when necessary. Parents and caregivers also require education and support to recognize that cyanosis and drowsiness in the presence of cough and rapid breathing are signs of severe pneumonia necessitating urgent medical attention. Provision of pulse oximeters to all health centers and remote nursing posts and training in their use must remain a priority.

## Contributors

KJB analyzed and interpreted the data, and wrote the first draft with guidance from HCM, L-AK, PCR, and CCB. CCB was the PNG lead investigator for both studies. JS, JK, BN and WK led recruitment and data management. RF, DL and WP led the design and on-site supervision of the first study. FMR was the lead researcher for the three country PneuCaPTIVE study. JC, RF and WP assisted with the design and implementation of the PneuCaPTIVE study. All authors had access to all the data in the study and accept responsibility to submit for publication. All authors reviewed the manuscript prior to publication.

## Data sharing statement

The study protocol and informed consent form are available upon request. Individual data from this study has not been made publicly available as data are only approved for use for the purposes outlined in the study protocol. Requests for de-identified data are subject to approval by the PNG Institute of Medical Research (PNGIMR) Institutional Review Board and PNG Medical Research Advisory Committee. We recommend that requests for data also be sent to Professor Chris Blyth.

## Declaration of interests

Investigators are supported by National Health and Medical Research Council Investigator grants and fellowships (CCB; FMR) and Perron Trust fellowships (HCM; LAK). The studies from which data were derived were funded by Pfizer Global and the Bill & Melinda Gates Foundation. PCR has served on scientific advisory boards on behalf of his institution for Pfizer, GlaxoSmithKline, Merck and AstraZeneca outside the scope of this study. HCM has served on scientific advisory boards on behalf of her institution for Pfizer, Sanofi, and Merck outside the scope of this study.
