# Supplementary material for: Clinical predictors of hypoxic pneumonia in children from the Eastern Highlands Province, Papua New Guinea: secondary analysis of two prospective observational studies
Source: Lancet Reg Health West Pac. 2024 Mar 27;45:101052. doi: 10.1016/j.lanwpc.2024.101052 (PMC11064719; doi:10.1016/j.lanwpc.2024.101052)
Supplement: Supplementary data [file mmc1.docx]

**Table 1. Key classifications used within this paper.**

| **Classification** | **Description** |
| --- | --- |
| WHO pneumonia^8^ | Cough and/or difficulty breathing plus fast breathing and/or chest indrawing. |
| WHO severe pneumonia^8^ | Cough and/or difficulty breathing plus any danger sign. |
| WHO danger signs^8^ | Inability to drink, persistent vomiting, convulsions, lethargy or unconsciousness, stridor in a calm child, severe malnutrition. |
| PNG mild pneumonia^11^ | Cough and tachypnoea (>60/min if <2 months, >50/min if 2-12 months, >40/min if >12 months of age). |
| PNG moderate pneumonia^11^ | Cough, tachypnoea (>60/min if <2 months, >50/min if 2-12 months, >40/min if >12 months of age), and chest indrawing. |
| PNG severe pneumonia^11^ | Cough, tachypnoea (>60/min if <2 months, >50/min if 2-12 months, >40/min if >12 months of age), and chest indrawing plus any of the following: heart failure (pulse >160/min with hepatomegaly >2 cm below costal margin), cyanosis or restlessness, or inability to breastfeed/drink, or vomiting. |
| WHO clinical signs to guide oxygen therapy in absence of pulse oximeter^9^ | Central cyanosis, inability to drink, severe chest indrawing, respiratory rate ≥70/min, grunting with every breath (in young infants), depressed mental status. |

**Table 2. Characteristics and clinical features of the study population: children aged 0-59 months presenting with moderate or severe pneumonia to health facilities in the Eastern Highlands Province between 2013 and 2019.**

| **Characteristics** | **Age <2 months**  **(n = 99); n (%)** | **Age 2-11 months**  **(n = 1151); n (%)** | **Age 12-59 months**  **(n = 797); n (%)** | **Total**  **(N = 2047); n (%)** |
| --- | --- | --- | --- | --- |
| **Demographic** | | | | |
| Female sex | 43 (43·4) | 503 (43·7) | 339 (42·5) | 885 (43·2) |
| **Risk factors** | | | | |
| Admitted in wet season^a^ | 43 (43·4) | 440 (38·2) | 328 (41·1) | 881 (39·6) |
| <1hr travel time to Goroka Town | 66 (66·7) | 775 (67·3) | 589 (73·9) | 1430 (73·9) |
| Malnutrition^b^ | 18 (18·2) | 215 (18·7) | 195 (24·5) | 424 (20·9) |
| Comorbid conditions^c^ | 2 (2·0) | 5 (0·4) | 10 (1·3) | 17 (0·8) |
| Adequately vaccinated^d^ | n/a | 365 (31·7) | 302 (37·9) | 667 (34·2) |
| **Clinical history (parent reported)** | | | | |
| Fever | 79 (79·8) | 1086 (94·3) | 757 (95·0) | 1922 (93·9) |
| Fast breathing | 99 (100·0) | 1115 (97·0) | 769 (96·5) | 1983 (96·9) |
| Apnoea or cyanosis | 12 (12·1) | 77 (6·7) | 24 (3·0) | 113 (5·5) |
| Difficulty feeding or drinking | 27 (27·3) | 204 (17·8) | 79 (9·9) | 310 (15·2) |
| Vomiting | 31 (31·1) | 196 (17·1) | 80 (10·0) | 307 (15·0) |
| Drowsiness | 5 (5·0) | 81 (7·1) | 51 (6·4) | 137 (6·7) |
| Irritability | 38 (38·4) | 563 (49·0) | 293 (36·8) | 894 (43·7) |
| Convulsions or seizures | 2 (2·0) | 28 (2·4) | 7 (0·9) | 37 (1·8) |
| **Physical examination (HCW assessed)** | | | | |
| Fever (≥38°C) | 15 (15·0) | 382 (33·2) | 302 (37·9) | 699 (34·1) |
| Age-specific tachycardia^e^ | 25 (25·5) | 247 (21·6) | 210 (26·4) | 482 (23·7) |
| Central cyanosis | 20 (20·8) | 143 (12·8) | 50 (6·4) | 213 (10·7) |
| Apnoea observed | 29 (30·2) | 223 (19·5) | 89 (11·2) | 341 (16·8) |
| Nasal discharge | 28 (28·3) | 653 (56·8) | 530 (66·5) | 1211 (59·2) |
| Severe lower chest indrawing | 21 (21·1) | 178 (15·5) | 40 (5·0) | 239 (11·7) |
| Nasal flaring or grunting | 16 (16·7) | 165 (14·8) | 55 (6·9) | 236 (11·8) |
| Delayed capillary refill (<3s) | 7 (7·1) | 49 (4·3) | 22 (2·8) | 78 (3·8) |
| Clinical dehydration^f^ | 9 (9·1) | 102 (8·9) | 57 (7·1) | 16 (8·2) |
| Abdominal distention | 2 (2·0) | 17 (1·5) | 97 (12·2) | 116 (5·7) |
| Pallor | 4 (4·1) | 13 (1·1) | 17 (2·1) | 34 (1·7) |
| Stridor | 4 (4·1) | 37 (3·3) | 21 (2·7) | 62 (3·1) |
| Wheeze | 16 (16·5) | 289 (25·5) | 189 (23·9) | 494 (24·5) |
| Crepitations | 92 (94·8) | 1126 (98·2) | 778 (98·7) | 1996 (98·3) |
| Bronchial breathing | 68 (71·6) | 828 (73·3) | 480 (61·1) | 1376 (68·5) |
| Reduced breath sounds | 68 (70·1) | 693 (60·8) | 412 (52·3) | 1173 (58·0) |
| Hepatomegaly | 1 (1·0) | 42 (3·7) | 18 (2·3) | 61 (3·0) |
| Decreased consciousness | 0 | 6 (0·5) | 4 (0·5) | 10 (0·5) |
| **Diagnosis and Outcomes** | | | | |
| PNG-defined moderate pneumonia^g^ | 70 (70·7) | 942 (81·8) | 754 (94·6) | 1766 (86·3) |
| PNG-defined severe pneumonia^g^ | 29 (29·3) | 209 (18·2) | 43 (5·4) | 281 (13·7) |
| Hypoxaemia (O_2_ saturation <90%) | 47 (47·5) | 451 (39·2) | 241 (30·2) | 739 (36·1) |
| Admitted to hospital | 61 (61·6) | 419 (36·4) | 143 (17·9) | 623 (30·4) |
| Bacteraemia^h^ | 0 | 25 (2·3) | 24 (3·2) | 49 (2·5) |
| In-hospital death | 1 (1·6) | 9 (2·1) | 2 (1·4) | 12 (1·9) |

^a^Wet season refers to period from December to April inclusive, dry season refers to period from May to November inclusive.

^b^Malnutrition defined as weight-for-age less than -2 z scores of median (WHO growth standards).

^c^Comorbid conditions: previously diagnosed conditions identified at the time of hospital presentation by parental report and review of handheld record.

^d^Adequately vaccinated: ≥2 doses of both DTPw-HepB-Hib and PCV13 in a child 2-11 months, ≥1 dose of both DTPw-HepB-Hib and PCV13 in a child ≥12 months. DTPw-HepB-Hib: diphtheria, tetanus, whole-cell pertussis, hepatitis B and *Haemophilus influenzae* type b vaccine. PCV13: 13-valent pneumococcal conjugate vaccine.

^e^Age-specific tachycardia: heart rate >160/min in a child <12 months, >120/min in a child aged 12 months to <5 years.^29^

^f^Signs of clinical dehydration include dry mucous membranes and/or reduced skin turgor.

^g^PNG-defined moderate pneumonia: cough, tachypnoea (respiratory rate ≥60/min if <2 months, ≥40/min if ≥2 months) and lower chest wall indrawing. PNG-defined severe pneumonia: moderate pneumonia plus heart failure (pulse >160/min with hepatomegaly >2 cm below costal margin), or cyanosis or restlessness, or inability to breastfeed/drink or vomiting.

^h^Bacteraemia: *Haemophilus influenzae*, *Streptococcus pneumoniae*, beta-haemolytic Streptococci, *Staphylococcus aureus* or Enterobacteriaceae spp. isolated from a blood culture.

HCW: Healthcare worker. Denominators vary due to missing data.

**Table 3. Characteristics and clinical features of hypoxaemic vs non-hypoxaemic children** **presenting with moderate or severe pneumonia to health facilities in the Eastern Highlands Province between 2013 and 2019, unadjusted analysis.**

| **Characteristic** | **Age <2 months** | | | **Age 2-11 months** | | | **Age 12-59 months** | | |
| --- | --- | --- | --- | --- | --- | --- | --- | --- | --- |
|  | **Hypoxaemic**  **n (%)**  **(n = 47)** | **Non-hypoxaemic n (%)**  **(n = 52)** | **Unadjusted OR**  **(95% CI)** | **Hypoxaemic**  **n (%)**  **(n = 451)** | **Non-hypoxaemic**  **n (%)**  **(n = 700)** | **Unadjusted OR**  **(95% CI)** | **Hypoxaemic**  **n (%)**  **(n = 241)** | **Non-hypoxaemic**  **n (%)**  **(n = 556)** | **Unadjusted OR**  **(95% CI)** |
| **Demographic** | | | | | | | | | |
| Female sex | 17 (36·2) | 26 (50·0) | 0·57  (0·25-1·27) | 203 (45·0) | 300 (42·9) | 1·09  (0·86-1·38) | 111 (46·1) | 228 (41·0) | 1·23  (0·90-1·66) |
| **Risk factors** | | | | | | | | | |
| Admitted in wet season | 18 (38·3) | 25 (48·1) | 0·67  (0·30-1·49) | 165 (36·6) | 275 (39·3) | 0·89  (0·69-1·14) | 84 (34·8) | 244 (43·9) | **0·68**  **(0·50-0·94)** |
| <1hr travel time to Goroka Town^a^ | 31 (66·0) | 35 (67·3) | 0·94  (0·41-2·17) | 318 (70·5) | 457 (65·3) | 1·27  (0·98-1·64) | 192 (79·7) | 397 (71·4) | **1·57**  **(1·09-2·26)** |
| Malnutrition^b^ | 10 (21·3) | 8 (15·4) | 1·48  (0·53-4·15) | 91 (20·2) | 124 (17·7) | 1·17  (0·87-1·59) | 64 (26·6) | 131 (23·6) | 1·17  (0·83-1·66) |
| Comorbid conditions^c^ | 2 (4·3) | 0 | - | 1 (0·2) | 4 (0·6) | 0·39  (0·04-3·47) | 3 (1·2) | 7 (1·3) | 0·98  (0·25-3·83) |
| Adequately vaccinated^d^ | - | - |  | 164 (36·4) | 201 (28·7) | **1·42**  **(1·10-1·82)** | 111 (46·1) | 191 (34·3) | **1·63**  **(1·20-2·22)** |
| **Clinical history (parent reported)** | | | | | | | | | |
| Fever | 40 (85·1) | 39 (75.0) | 1·90  (0·69-5·27) | 428 (94·9) | 658 (94·0) | 1·19  (0·70-2·00) | 234 (97·1) | 523 (94·1) | 2·11  (0·92-4·84) |
| Fast breathing | 47 (100·0) | 52 (100·0) | - | 443 (98·2) | 672 (96·1) | **2·22**  **(1·00-4·94)** | 235 (97·5) | 534 (96·0) | 1·61  (0·65-4·03) |
| Apnoea or cyanosis | 10 (21·3) | 2 (3·8) | **6·76**  **(1·39-32·69)** | 43 (9·6) | 34 (4·9) | **2·06**  **(1·29-3·28)** | 15 (6·2) | 9 (1·62) | **4·03**  **(1·74-9·33)** |
| Difficulty feeding or drinking | 20 (42·5) | 7 (13·5) | **4·76**  **(1·78-12·74)** | 105 (23·3) | 99 (14·2) | **1·84**  **(1·35-2·49)** | 39 (16·2) | 40 (7·2) | **2·48**  **(1·55-3·98)** |
| Vomiting | 19 (40·4) | 12 (23·1) | 2·26  (0·95-5·39) | 89 (19·2) | 107 (15·3) | **1·36**  **(1·00-1·86)** | 32 (13·3) | 48 (8·6) | **1·62**  **(1·01-2·61)** |
| Drowsiness | 3 (6·4) | 2 (3·8) | 1·70  (0·27-10·67) | 46 (10·2) | 35 (5·0) | **2·15**  **(1·36-3·39)** | 22 (9·1) | 29 (5·2) | **1·82**  **(1·02-3·24)** |
| Irritability | 14 (29·8) | 24 (46·1) | 0·49  (0·22-1·13) | 242 (53·8) | 321 (45·9) | **1·37**  **(1·08-1·74)** | 117 (48·5) | 176 (31·7) | **2·03**  **(1·49-2·77)** |
| Convulsions or seizures | 2 (4·3) | 0 | - | 12 (2·7) | 16 (2·3) | 1·16  (0·54-2·48) | 1 (0·4) | 6 (1·1) | 0·38  (0·05-3·18) |
| **Physical examination (HCW assessed)** | | | | | | | | | |
| Fever (≥38°C) | 9 (19·1) | 6 (11·5) | 1·82  (0·59-5·56) | 160 (35·5) | 222 (31·7) | 1·18  (0·92-1·52) | 107 (44·4) | 195 (35·1) | **1·48**  **(1·09-2·01)** |
| Age-specific tachycardia^e^ | 15 (31·9) | 10 (19·6) | 1·92  (0·76-4·84) | 147 (32·7) | 100 (14·4) | **2·88**  **(2·16-3·85)** | 103 (42·9) | 107 (19·3) | **3·14**  **(2·25-4·37)** |
| Central cyanosis | 18 (40·0) | 2 (3·9) | **16·3**  **(3·5-75·77)** | 112 (25·7) | 31 (4·6) | **7·25**  **(4·76-11·03)** | 36 (15·2) | 14 (2·6) | **6·84**  **(3·61-12·95)** |
| Apnoea observed | 22 (47·8) | 7 (14·0) | **5·63**  **(2·10-15·10)** | 133 (29·7) | 90 (12·9) | **2·84**  **(2·11-3·84)** | 45 (18·7) | 44 (7·9) | **12·66**  **(1·70-4·16)** |
| Nasal discharge | 10 (21·3) | 18 (34·6) | 0·51  (0·21-1·26) | 246 (54·5) | 407 (58·3) | 0·86  (0·67-1·09) | 145 (60·2) | 385 (69·2) | **0·67**  **(0·49-0·92)** |
| Severe lower chest indrawing | 14 (30·4) | 7 (13·5) | **2·81**  **(1·02-7·75)** | 114 (25·3) | 64 (9·2) | **3·35**  **(2·40-4·68)** | 24 (10·0) | 16 (2·9) | **3·72**  **(1·94-7·15)** |
| Nasal flaring or grunting | 14 (30·4) | 2 (4·0) | **10·5**  **(2·23-49·35)** | 122 (27·8) | 43 (6·3) | **5·67**  **(3·91-8·24)** | 36 (14·9) | 19 (3·4) | **4·93**  **(2·76-8·79)** |
| Delayed capillary refill (<3s) | 6 (12·8) | 1 (1·9) | 7·46  (0·86-64·49) | 31 (6·9) | 18 (2·6) | **2·8**  **(1·53-5·02)** | 10 (4·1) | 12 (2·2) | 1·96  (0·83-4·60) |
| Clinical dehydration^f^ | 5 (10·6) | 4 (7·7) | 1·43  (0·36-5·67) | 40 (8·9) | 62 (8·9) | 1·00  (0·66-1·52) | 17 (7·0) | 40 (7·2) | 0·98  (0·54-1·76) |
| Abdominal distention | 1 (2·1) | 1 (1·9) | 1·12  (0·07-18·24) | 9 (2·0) | 8 (1·1) | 1·76  (0·67-4·60) | 14 (5·8) | 83 (14·9) | **0·35**  **(0·19-0·63)** |
| Pallor | 3 (6·4) | 1 (2·0) | 3·41  (0·34-33·97) | 8 (1·8) | 5 (0·7) | 2·51  (0·81-7·72) | 6 (2·49) | 11 (1·98) | 1·26  (0·46-3·45) |
| Stridor | 3 (6·5) | 1 (1·9) | 3·56  (0·36-35·46) | 26 (5·9) | 11 (1·6) | **3·81**  **(1·86-7·79)** | 17 (7·1) | 4 (0·7) | **10·35**  **(3·44-31·10)** |
| Wheeze | 9 (19·6) | 7 (13·7) | 1·53  (0·52-4·50) | 148 (33·0) | 141 (20·6) | **1·90**  **(1·45-2·48)** | 91 (37·9) | 98 (17·8) | **2·81**  **(2·00-3·95)** |
| Crepitations | 45 (95·7) | 47 (94·0) | 1·44  (0·23-9·00) | 448 (99·6) | 678 (97·4) | **5·95**  **(1·37-25·75)** | 238 (99·6) | 540 (98·4) | 3·97  (0·50-31·48) |
| Bronchial breathing | 41 (87·2) | 27 (56·2) | **5·31**  **(1·90-14·87)** | 362 (80·6) | 466 (68·5) | **1·91 (1·44-2·54)** | 191 (79·6) | 289 (53·0) | **3·45**  **(2·42-4·93)** |
| Reduced breath sounds | 38 (80·8) | 30 (60·0) | **2·81**  **(1·12-7·07)** | 343 (76·2) | 350 (50·8) | **3·10**  **(2·38-4·04)** | 181 (75·4) | 231 (42·2) | **4·20**  **(3·00-5·89)** |
| Hepatomegaly | 1 (2·1) | 0 | - | 29 (6·5) | 13 (1·9) | **3·59**  **(1·85-7·00)** | 12 (5·0) | 6 (1·1) | **4·77**  **(1·77-12·90)** |
| Decreased consciousness | 0 | 0 | - | 4 (0·9) | 2 (0·3) | 3·12  (0·57-17·10) | 2 (0·8) | 2 (0·4) | 2·31  (0·32-16·49) |

^a^Wet season refers to period from December to April inclusive, dry season refers to period from May to November inclusive.

^b^Malnutrition defined as weight-for-age less than -2 z scores of median (WHO growth standards).

^c^Comorbid conditions: previously diagnosed conditions identified at the time of hospital presentation by parental report and review of handheld record.

^d^Adequately vaccinated: ≥2 doses of both DTPw-HepB-Hib and PCV13 in a child 2-11 months, ≥1 dose of both DTPw-HepB-Hib and PCV13 in a child ≥12 months. DTPw-HepB-Hib: diphtheria, tetanus, whole-cell pertussis, hepatitis B and *Haemophilus influenzae* type b vaccine. PCV13: 13-valent pneumococcal conjugate vaccine.

^e^Age-specific tachycardia: heart rate >160/min in a child <12 months, >120/min in a child aged 12 months to <5 years.^29^

^f^Signs of clinical dehydration include dry mucous membranes and/or reduced skin turgor.

HCW: Healthcare worker. Denominators vary due to missing data.

**Table 4. Multivariable analysis of risk factors and clinical features associated with hypoxic pneumonia in children presenting with moderate or severe pneumonia to health facilities in the Eastern Highlands Province between 2013 and 2019**

| **Potential predictors of hypoxaemia** | | **Adjusted OR (95% CI)** | | | |
| --- | --- | --- | --- | --- | --- |
|  | | **Age <2 months** | **Age 2-11 months** | **Age 12-59 months** | **All ages** |
| Age | |  |  |  |  |
|  | <2 months | - | - | - | 2·32 (1·38-3·90) |
|  | 2-11 months | - | - | - | 1·35 (1·07-1·69) |
|  | 12-59 months | - | - | - | 1 |
| Presented in wet season | | 0·52 (0·18-1·49) | 0·82 (0·61-1·09) | 0·56 (0·38-0·81) | 0·71 (0·57-0·89) |
| Proximity to hospital (<1hr) | | 1·13 (0·35-3·59) | 1·54 (1·12-2·11) | 1·58 (1·02-2·47) | 1·55 (1·21-1·99) |
| Adequately vaccinated^a^ | | - | 1·81 (1·32-2·46) | 2·05 (1·39-3·03) | 1·94 (1·52-2·46) |
| Drowsiness (parent reported) | | 0·44 (0·01-14·59) | 1·89 (1·08-3·32) | 1·61 (0·79-3·28) | 1·75 (1·14-2·69) |
| Irritability (parent reported) | | 0·46 (0·15-1·41) | 1·08 (0·81-1·45) | 1·51 (1·03-2·21) | 1·18 (0·94-1·48) |
| Age-specific tachycardia^b^ | | 1·40 (0·36-5·43) | 1·92 (1·34-2·73) | 1·90 (1·28-2·84) | 1·89 (1·46-2·44) |
| Central cyanosis (on examination) | | 9·47 (1·57-56·96) | 5·27 (3·25-8·56) | 4·68 (2·21-9·91) | 5·14 (3·47-7·60) |
| Nasal flaring or grunting | | 6·19 (0·94-40·93) | 2·50 (1·59-3·93) | 1·82 (0·91-3·63) | 2·34 (1·62-3·38) |
| Wheezing | | 0·51 (0·10-2·61) | 1·68 (1·21-2·34) | 2·16 (1·42-3·30) | 1·76 (1·36-2·26) |
| Bronchial breathing | | 3·87 (1·04-14·44) | 1·06 (0·74-1·51) | 1·92 (1·23-2·98) | 1·42 (1·09-1·85) |
| Reduced breath sounds | | 1·66 (0·50-5·50) | 2·76 (2·02-3·77) | 3·39 (2·26-5·08) | 2·92 (2·30-3·71) |

^a^Adequately vaccinated: ≥2 doses of both DTPw-HepB-Hib and PCV13 in a child 2-11 months, ≥1 dose of both DTPw-HepB-Hib and PCV13 in a child ≥12 months. DTPw-HepB-Hib: diphtheria, tetanus, whole-cell pertussis, hepatitis B and *Haemophilus influenzae* type b vaccine. PCV13: 13-valent pneumococcal conjugate vaccine.

^b^Age-specific tachycardia: heart rate >160/min in a child <12 months, >120/min in a child aged 12 months to <5 years.^29^

**Table 5. Validity of the developed model and existing definitions for detecting hypoxaemia in children from the present study.**

| **Model** | **Age group** | **Sensitivity** | **Specificity** | **PPV** | **NPV** | **LR+** | **LR-** | **AUC** |
| --- | --- | --- | --- | --- | --- | --- | --- | --- |
| **Combined model (developed on this dataset)*** | Overall | 53% | 88% | 72% | 75% | 4·46 | 0·54 | 0·781 |
|  | 0-2m | 69% | 87% | 84% | 74% | 5·17 | 0·36 | 0·834 |
|  | 2-11m | 51% | 88% | 74% | 73% | 4·29 | 0·55 | 0·769 |
|  | 1-5y | 44% | 92% | 70% | 79% | 5·34 | 0·61 | 0·795 |
| **PNG severe pneumonia^11^** | Overall | 25% | 96% | 76% | 69% | 5·78 | 0·80 | 0·615 |
|  | 0-2m | 58% | 84% | 76% | 69% | 3·68 | 0·50 | 0·731 |
|  | 2-11m | 26% | 95% | 76% | 67% | 4·93 | 0·78 | 0·615 |
|  | 1-5y | 14% | 97% | 70% | 73% | 5·53 | 0·88 | 0·579 |
| **WHO severe pneumonia^8^** | Overall | 17% | 94% | 63% | 66% | 2·92 | 0·88 | 0·597 |
|  | 0-2m | 43% | 86% | 73% | 64% | 3·14 | 0·66 | 0·664 |
|  | 2-11m | 21% | 90% | 59% | 64% | 2·18 | 0·88 | 0·580 |
|  | 1-5y | 11% | 98% | 67% | 72% | 5·36 | 0·91 | 0·602 |
| **WHO clinical signs to guide oxygen therapy in absence of pulse oximeter^9^** | Overall | 45% | 87% | 66% | 74% | 3·45 | 0·63 | 0·673 |
|  | 0-2m | 59% | 94% | 90% | 72% | 9·85 | 0·44 | 0·762 |
|  | 2-11m | 47% | 85% | 67% | 71% | 3·09 | 0·62 | 0·675 |
|  | 1-5y | 31% | 93% | 67% | 75% | 4·62 | 0·74 | 0·648 |

PPV = Positive Predictive Value; NPV = Negative Predictive Value; LR+ positive likelihood ratio (LR+ = sensitivity/(1-specificity)); LR- negative likelihood ratio (LR- = specificity/(1-sensitivity)); AUC = the area under the ROC curve. A positive LR >10 and a negative LR <0.1 are considered to exert highly significant changes in probability, such as to alter clinical management.^30^

* Presented in wet season^a^, proximity to hospital (<1hr), adequately vaccinated^b^, history of drowsiness,history of irritability, tachycardia^d^, central cyanosis, nasal flaring or grunting, wheezing, bronchial breathing, reduced breath sounds ^a^Wet season refers to period from December to April inclusive, dry season refers to period from May to November inclusive.

^b^Adequately vaccinated: ≥2 doses of both DTPw-HepB-Hib and PCV13 in a child 2-11 months, ≥1 dose of both DTPw-HepB-Hib and PCV13 in a child ≥12 months. DTPw-HepB-Hib: diphtheria, tetanus, whole-cell pertussis, hepatitis B and *Haemophilus influenzae* type b vaccine. PCV13: 13-valent pneumococcal conjugate vaccine.

^c^Signs of clinical dehydration include dry mucous membranes and/or reduced skin turgor.

^d^Age-specific tachycardia: heart rate >160/min in a child <12 months, >120/min in a child aged 12 months to 4 years.^29^

**Supplementary materials**

**Supplementary Table 1. Multivariable analyses of risk factors and clinical features associated with hypoxic pneumonia in children presenting with moderate or severe pneumonia to health facilities in the Eastern Highlands Province between 2013 and 2019.****Logistic regression models were developed separately for children aged <2 months (1a), 2-11 months (1b), 12-59 months (1c), and all children (1d).**

**Supplementary Table 1a**

| **Potential predictors of hypoxaemia** | **Adjusted OR (95% CI)** | **p-value** |
| --- | --- | --- |
| Presented in wet season^a^ | 0·36 (0·11-1·14) | 0·083 |
| Irritability (parent reported) | 0·28 (0·08-1·01) | 0·052 |
| Vomiting (parent reported) | 5·52 (1·54-19·79) | 0·009 |
| Central cyanosis (on examination) | 12·29 (1·86-81·11) | 0·009 |
| Bronchial breathing | 5·71 (1·42-22·99) | 0·014 |

**Supplementary Table 1b**

| **Potential predictors of hypoxaemia** | **Adjusted OR (95% CI)** | **p-value** |
| --- | --- | --- |
| Proximity to hospital (<1hr) | 1·48 (1·06-2·06) | 0·020 |
| Adequately vaccinated^b^ | 1·67 (1·23-2·27) | 0·001 |
| Drowsiness (parent reported) | 1·85 (1·00-3·41) | 0·048 |
| Age-specific tachycardia (>160bpm) | 1·95 (1·36-2·79) | <0·001 |
| Central cyanosis (on examination) | 4·95 (3·03-8·09) | <0·001 |
| Nasal flaring or grunting | 2·67 (1·66-4·28) | <0·001 |
| Clinical dehydration^c^ | 0·61 (0·35-1·04) | 0·069 |
| Wheezing | 1·78 (1·28-2·49) | 0·001 |
| Crepitations | 7·25 (0·89-59·04) | 0·064 |
| Reduced breath sounds | 2·94 (2·16-3·99) | <0·001 |

**Supplementary Table 1c**

| **Potential predictors of hypoxaemia** | **Adjusted OR (95% CI)** | **p-value** |
| --- | --- | --- |
| Presented in wet season^a^ | 0·57 (0·39-0·83) | 0·004 |
| Proximity to hospital (<1hr) | 1·49 (0·96-2·32) | 0·076 |
| Adequately vaccinated^b^ | 2·14 (1·44-3·18) | <0·001 |
| Irritability (parent reported) | 1·57 (1·07-2·31) | 0·022 |
| Age-specific tachycardia^d^ | 1·83 (1·22-2·74) | 0·003 |
| Central cyanosis(on examination) | 4·91 (2·29-10·55) | <0·001 |
| Nasal discharge | 0·70 (0·47-1·05) | 0·082 |
| Nasal flaring or grunting | 1·92 (0·95-3·88) | 0·069 |
| Wheezing | 2·33 (1·52-3·56) | <0·001 |
| Bronchial breathing | 1·73 (1·10-2·73) | 0·018 |
| Reduced breath sounds | 3·48 (2·31-5·23) | <0·001 |

**Supplementary Table 1d**

| **Potential predictors of hypoxaemia** | **Adjusted OR (95% CI)** | **p-value** |
| --- | --- | --- |
| Presented in wet season^a^ | 0·70 (0·56-0·88) | 0·002 |
| Proximity to hospital (<1hr) | 1·48 (1·15-1·90) | 0·002 |
| Adequately vaccinated^b^ | 1·86 (1·47-2·37) | <0·001 |
| Drowsiness (parent reported) | 1·55 (0·98-2·44) | 0·059 |
| Age-specific tachycardia^d^ | 1·77 (1·37-2·29) | <0·001 |
| Central cyanosis (on examination) | 4·50 (2·97-6·80) | <0·001 |
| Apnoea observed | 1·53 (1·09-2·14) | 0·013 |
| Nasal flaring or grunting | 2·17 (1·47-3·22) | <0·001 |
| Wheezing | 1·80 (1·40-2·32) | <0·001 |
| Bronchial breathing | 1·39 (1·06-1·82) | 0·017 |
| Reduced breath sounds | 3·33 (2·60-4·25) | <0·001 |

^a^Wet season refers to period from December to April inclusive, dry season refers to period from May to November inclusive

^b^Adequately vaccinated: ≥2 doses of both DTPw-HepB-Hib and PCV13 in a child 2-11 months, ≥1 dose of both DTPw-HepB-Hib and PCV13 in a child ≥12 months. DTPw-HepB-Hib: diphtheria, tetanus, whole-cell pertussis, hepatitis B and *Haemophilus influenzae* type b vaccine. PCV13: 13-valent pneumococcal conjugate vaccine.

^c^Signs of clinical dehydration include dry mucous membranes and/or reduced skin turgor.

^d^Age-specific tachycardia: heart rate >160/min in a child <12 months, >120/min in a child aged 12 months to <5 years.^29^

**Supplementary Table 2. Validity of the multivariable logistic regression models to detect hypoxaemia in children presenting with moderate or severe pneumonia to health facilities in the Eastern Highlands Province between 2013 and 2019. Presented are the age group specific model (2a) and the combined model (2b), by age group.**

**Supplementary Table 2a**

| **Age group** | **Predictors** | **Sensitivity** | **Specificity** | **LR+** | **LR-** | **AUC** |
| --- | --- | --- | --- | --- | --- | --- |
| <2m | Presented in wet season^a^, history of vomiting, history of irritability, central cyanosis, bronchial breathing | 74% | 74% | 2·88 | 0·35 | 0·849 |
| 2-11m | Proximity to hospital (<1hr), adequately vaccinated^b^, history of drowsiness, tachycardia (>160bpm), central cyanosis, nasal flaring or grunting, clinical dehydration^c^, wheezing, crepitations, reduced breath sounds | 53% | 87% | 4·13 | 0·53 | 0·772 |
| 12-59m | Presented in wet season^a^, proximity to hospital (<1hr), adequately vaccinated^b^, history of irritability, tachycardia (>120bpm), central cyanosis, nasal discharge, nasal flaring or grunting, wheezing, bronchial breathing, reduced breath sounds | 47% | 90% | 4·93 | 0·59 | 0·799 |
| 0-59m | Presented in wet season^a^, proximity to hospital (<1hr), adequately vaccinated^b^,  history of drowsiness, history of irritability, tachycardia^d^, central cyanosis,  nasal flaring or grunting, wheezing,  bronchial breathing, reduced breath sounds | 53% | 88% | 4·46 | 0·54 | 0·781 |

**Supplementary Table 2b**

| **Age group** | **Predictors** | **Sensitivity** | **Specificity** | **LR+** | **LR-** | **AUC** |
| --- | --- | --- | --- | --- | --- | --- |
| <2m | Presented in wet season^a^, proximity to hospital (<1hr), adequately vaccinated^b^,  history of drowsiness, history of irritability, tachycardia^d^, central cyanosis,  nasal flaring or grunting, wheezing,  bronchial breathing, reduced breath sounds | 69% | 87% | 5·17 | 0·36 | 0·834 |
| 2-11m |  | 51% | 88% | 4·29 | 0·55 | 0·769 |
| 12-59m |  | 44% | 92% | 5·34 | 0·61 | 0·796 |
| 0-59m |  | 53% | 88% | 4·46 | 0·54 | 0·781 |

^a^Wet season refers to period from December to April inclusive, dry season refers to period from May to November inclusive.

^b^Adequately vaccinated: ≥2 doses of both DTPw-HepB-Hib and PCV13 in a child 2-11 months, ≥1 dose of both DTPw-HepB-Hib and PCV13 in a child ≥12 months. DTPw-HepB-Hib: diphtheria, tetanus, whole-cell pertussis, hepatitis B and *Haemophilus influenzae* type b vaccine. PCV13: 13-valent pneumococcal conjugate vaccine.

^c^Signs of clinical dehydration include dry mucous membranes and/or reduced skin turgor.

^d^Age-specific tachycardia: heart rate >160/min in a child <12 months, >120/min in a child aged 12 months to <5 years.^29^

LR+ positive likelihood ratio (LR+ = sensitivity/(1-specificity)); LR- negative likelihood ratio (LR- = specificity/(1-sensitivity)); AUC = the area under the ROC curve. A positive LR >10 and a negative LR <0.1 are considered to exert highly significant changes in probability, such as to alter clinical management.^30^
